# Perinatal Factors Leading to Birth Asphyxia among Term Newborns in a Tertiary Care Hospital

**Published:** 2014-09-12

**Authors:** Asad Nauman Kiyani, Arshad Khushdil, Azra Ehsan

**Affiliations:** Department of Pediatrics, Combined Military Hospital Multan, Pakistan

**Keywords:** Birth Asphyxia, Perinatal Risk Factors, Frequency, Meconium Aspiration, Pakistan

## Abstract

***Objective:*** To determine various perinatal factors leading to birth asphyxia among term newborns in a tertiary care hospital.

***Methods:*** In a cross sectional study, a total of 196 asphyxiated cases were selected through consecutive non-probability sampling technique from neonatal intensive care unit (NICU) of a tertiary care Military Hospital in Pakistan from 1st December 2012 to 1st December 2013. Data obtained was analyzed using SPSS version 15.0. Descriptive statistics were used to calculate means, standard deviations and frequencies. Stratification with respect to maternal age, gestational age, newborns weight, parity and gravidity was done and post stratification chi-square test was applied to find statistical significance.

***Findings:*** Out of 196 cases, 125 (64%) were males and 71 females (36%). Mean maternal age was 27.04+4.97 years and gestational age of babies was 39.86+1.24 weeks. Majority (57.14 %) of 112 mothers were 1-3 para and ≥4 parity was recorded in 84 (42.86%) cases. Majority (64.80%) of the 127 mothers were 1-3 gravida while 69 (35.20%) had ≥4 gravidity, mean of 3.45+0.87. Mode of delivery as a factor leading to birth asphyxia was found in 32.14% (n=63) cesarean section, 44.39% (n=87) spontaneous vertex delivery, and instrumental delivery in 23.47% (n= 46). Prolonged second stage of labor reported in 72% (n=141), 29.08% (n=57) had prolonged rupture of membranes, 7.65% (n=15) had meconium staining, 5.61% (n=11) had multiple births, 21.94% (n=43) had maternal fever, and 58.84% (n=113) had anemia at delivery.

***Conclusion:*** Birth asphyxia is a preventable problem and long term neurological sequelae almost untreatable. Timely identification of the perinatal risk factors and their prompt solution can prevent and reduce the neonatal morbidity and mortality from birth asphyxia. Early identification of high-risk cases with improved antenatal and perinatal care can further decrease such high mortality.

## Introduction

Birth asphyxia is a leading cause of mortality and morbidity in neonates in developing countries, with an incidence of 100-250/1000 live births compared to 5-10/1000 live births in the developed world^[^^1^^]^. It remains a significant cause of loss of life and adverse developmental outcome^[^^2^^]^. The major causes of neonatal deaths globally were estimated to be infections (35%), preterm births (28%) and birth asphyxia (23%)^[^^3^^]^.

 As large number of deliveries in the developing world takes place at home, there is no reliable data to precisely estimate the disease burden in countries like Pakistan. However, this figure is likely to be very high as two thirds of world’s neonatal mortality occurs in 10 developing countries^[^^4^^]^. In Pakistan, over 5 million children are born each year. Of them 0.45 million die before first birthday and nearly half of these deaths occur during the neonatal period^[^^5^^]^. In 2001, birth asphyxia was responsible for 35%, 14% and 11% of neonatal mortality in Lahore, Karachi and Khyber Pukhtunkhwa respectively^[^^6^^]^.

 Various risk factors are associated with birth asphyxia. Though the topic has been extensively studied and reviewed worldwide, limited local data is available; Common intrapartum risk factors in a local data include non cephalic presentation, prolonged rupture of membranes, meconium staining, maternal anemia, vaginal bleeding, maternal fever at time of delivery, mode of delivery after prolonged second stage of labor like normal vertex delivery, cesarean section, and multiple births^[^^5^^,^^6^^]^. It is estimated that for every 1 death due to birth asphyxia 4 infants survive with long-term sequel^[^^6^^]^. 

 The American Academy of Pediatrics and a task force on cerebral Palsy has suggested criterion to define birth asphyxia^[^^7^^]^: 

(1) Profound metabolic or mixed metabolic acidemia of pH <7.00 in an umbilical artery blood sample taken at birth. 

(2) Persistence of an Apgar score of 0-3 for ≥5 minutes. 

(3) Neonatal neurologic manifestations e.g. seizures, coma or hypotonia following an asphyxia insult. 

(4) Evidence of multi-organ involvement e.g. cardiovascular, gastrointestinal, or renal compromise. 

 There is nothing more tragic for a normally developed fetus to sustain cerebral anoxia during the last hours of perinatal life due to perinatal risk factors thus resulting in death or if escaped, has to live with major handicap. This study was planned with the aim to find out the leading perinatal risk factors in term babies causing birth asphyxia in our setup.

## Subjects and Methods

This descriptive–cross sectional study was carried out at the neonatal intensive care unit (NICU) of Combined Military Hospital, Multan Pakistan, a tertiary care hospital from 1st December 2012 to 1st December 2013. Total of 196 asphyxiated cases that fulfilled the inclusion and exclusion criteria were included in the study by taking p 4.8% and d 3%^[^^1^^]^ through consecutive non-probability sampling technique.

*Inclusion Criteria:*

• Neonates suffering from perinatal asphyxia diagnosed in the presence of at least 2 of the following factors, and admitted to neonatal intensive care unit within 6 hours of birth.

• First cry delayed for 5 minutes. 

• Apgar score at 5 minutes of age <5 and didn’t improve to more than 7/10 at 20 minutes of age.

• Post asphyxial seizures within first 48 hours after birth.

*Exclusion Criteria:*

• Neonates with major congenital malformations of central nervous system, cardiovascular system, respiratory system or dysmorphic babies.

• babies delivered by lower segment cesarean section (LSCS) to mothers who were given general anesthesia

• Term intra-uterine growth retardation (IUGR) babies with birth weight less than 1.5 kg.

• Preterm deliveries.

• Other causes of central nervous system encephalopathy (infectious, metabolic).

• All outdoor deliveries.

 Approval from hospital ethical committee was sought. Informed written consent was taken from all the patients’ parents participating in this study. All neonates admitted to neonatal intensive care unit (NICU) and fulfilling the inclusion criteria were taken as subjects of the study. Every baby was assigned a serial number. Detailed history was taken from mother and birth attendant (gynaecologist/ gynae nurse) on a predesigned questionnaire regarding selected perinatal factors (mode of delivery (spontaneous vertex delivery, cesarean section, instrumental delivery), abnormal presentation, rupture of membranes more than 18 hours, prolonged second stage of labor more than 30 minutes, maternal fever and anemia at the time of delivery, and meconium staining). Babies were examined in detail for the signs of birth asphyxia (tone, posture, reflexes, seizures etc). Data obtained was analyzed using SPSS version 15.0. Descriptive statistics were used to calculate mean, and standard deviation was used for gestational age, parity, maternal age, gravidity, and weight of the newborns. Frequencies and percentages were calculated for variables like mode of delivery, instrumental delivery, maternal complications like fever and anemia at delivery, prolonged rupture of membranes, meconium staining and multiple births. Stratification with respect to maternal age, gestational age, and weight of newborns, parity and gravidity was done and post stratification chi-square test was applied. *P-*value <0.05 was taken as significant.

## Findings

A total of 196 cases fulfilling the inclusion/exclusion criteria were enrolled in the study, 125 (64%) were males and 71 (36%) females. Maternal age of the patients showed 54.59% (n=107) between 18-27 years and 45.41% (n=89) between 28-37 years, mean and standard deviation was calculated as 27.04 (±4.97) years. Majority (52.55%) of babies (n=103) were born between 37-39 weeks while 47.45 % (n=93) between 40-41 weeks of gestation, mean and standard deviation was calculated as 39.19 (+1.24) weeks. Majority (57.14 %, n=112) of mothers were between 1-3 and ≥4 paras and were recorded in 42.86 % (n=84) cases, mean and standard deviation 2.87 (+1.12). Most of the mothers were between 1-3 gravida, i.e. 64.80% (n=127) while 35.20 % (n=69) had ≥4 gravidities, mean and standard deviation was 3.45 (+0.87). Mean weight of the newborns was calculated as 2621.37 (+74.21) grams.

 Frequency of perinatal factors leading to birth asphyxia among term newborns were evaluated; prolonged second stage of labor reported in 141 (72%) cases, mode of delivery was spontaneous vertex delivery in 44.39% (n=87), cesarean section in 32.14% (n=63), 23.47% (n= 46) instrumental delivery. Fifty-seven patients (29.08%) had prolonged rupture of membranes (PROM), 7.65% (n=15) had meconium staining, 5.61% (n=11) had multiple births, 21.94% (n=43) had maternal fever, 58.84% (n=113) had anemia at delivery.

 Stratification with regards to maternal age was recorded which shows anemia at delivery was significantly higher in 28-37 years of age, *P *value was calculated as 0.0001 while rest of risk factors were insignificant (Table 1).

 Stratification with regards to gestational age was recorded which shows that each factor was significantly higher in gestational age between 40-41 weeks of gestation as compared to 37-39 weeks (Table 2).

 In Table 3, stratification with regards to parity was computed where instrumental delivery, prolonged rupture of membranes and maternal fever was insignificant in both groups while others i.e. cesarean section, spontaneous vertex delivery, meconium staining and anemia at delivery had significant difference between 1-3 and ≥4 paras.

## Discussion

This study was planned to determine the various perinatal risk factors associated with birth asphyxia in term newborns in neonatal intensive care unit. As 23% of all neonatal deaths are attributable to birth asphyxia^[^^2^^]^, it is important to be aware of factors that may predispose a newborn to a hypoxic insult at birth with the aim of formulating preventive strategies.

**Table 1:** Stratification for maternal age (n=196)

**Factors**	**Maternal age (years), n (%)**		***P. *** **value**
	**18-27 years**	**28-37 years**	
**Instrumental delivery (n=46)**	21 (45.65%)	25 (54.35%)	0.2
**Spontaneous vertex delivery (n=87)**	49 (56.32%)	38 (43.68%)	0.7
**Cesarean section (n=63)**	34 (53.97%)	29 (46.03%)	0.9
**Prolonged rupture of membranes (n=57)**	26 (45.61%)	31 (54.39%)	0.1
**Meconium staining (n=15)**	6 (40%)	9 (60%)	0.2
**Maternal Fever (n=43)**	26 (60.47%)	27 (62.79%)	0.3
**Anemia at delivery (n=113)**	45 (40.46%)	68 (59.54%)	0.0001

**Table 2:** Stratification for gestational age (n=196)

**Factors**	**Gestational age (years), n (%)**		***P. *** **value**
	**37-39 weeks ** **(n=103)**	**40-41 weeks ** **(n=93)**	
**Instrumental delivery (n=46)**	15 (32.61)	31 (67.39)	0.002
**Spontaneous vertex delivery (n=87)**	33 (37.93)	54 (62.07)	0.001
**Cesarean section (n=63)**	43 (68.25)	20 (31.75)	0.002
**Prolonged rupture of membranes (n=57)**	19 (33.33)	38 (66.67)	0.001
**Meconium staining (n=15)**	3 (20)	12 (80)	0.008
**Maternal fever (n=43)**	12 (27.91)	31 (72.09)	0.001
**Anemia at delivery (n=113)**	33 (28.24)	80 (71.76)	0.001

These factors may be antepartum in 50% of cases, intrapartum in 40% and postpartum in remaining 10%. Given the reduced availability of skilled care during delivery in developing countries like Pakistan, intrapartum causes may have greater contribution^[^^6^^]^. 

 In this study full term neonates (>1.5kg and >37 weeks of gestation) of hospital deliveries admitted in the neonatal intensive care unit were included, male to female distribution was 3:2. Out of 196 cases 64% were males, Ibrahim^[^^13^^]^ et al in their study identified weight of more than 2.5 kg and full term male neonates as risk factors leading to birth asphyxia in an analysis of 235 cases of birth asphyxia, as in our study. 

 Prolonged second stage of labor (>30 mins) remained the most important determinant of birth asphyxia as in other studies as well as mode of delivery^[^^8^^,^^12^^]^, Spontaneous vertex delivery (SVD) was the most common mode of delivery in our study associated with birth asphyxia in 87 (44.39%), followed by emergency cesarean section (CS) in 63 (32.14%), and instrumental delivery (included both forceps and vacuum delivery) in 46 (23.47%). This is similar to other studies like Chishty^[^^12^^]^ et al where proportion of birth asphyxia was predominantly greater in spontaneous vertex delivery (SVD) in in-hospital cases, but Zulfiqar^[^^14^^]^ et al in their study described 66% of cases associated with birth asphyxia delivered by cesarean section and 34% with spontaneous vertex delivery in in-hospital study.

 We had only 3 cases of birth asphyxia followed by elective cesarean section, which is significantly lower number. Different studies also describe this inverse association between elective cesarean section and birth asphyxia^5^. In recent years, the rate of cesarean section has risen to a record level of 46% in China and to levels of 25% and above in many Asian, European and Latin American countries^[^^9^^]^. The rate has increased significantly in the United States, to 33 percent of all births in 2011, up from 21 percent in 1996, and in the rate in 2009 varied widely between hospitals (ranging from 6.9% to 69.9% of births)^[^^10^^]^. Higher number of CS in in-cases shows skills of detecting perinatal asphyxia in a tertiary care hospital and going for early intervention.

**Table 3:** Stratification for parity (n=196)

**Factors**	**Parity n (%)**		***P*** **-value**
	**1-3 (n=112)**	**≥4 (n=84)**	
**Instrumental delivery (n=46)**	11 (23.91)	35 (76.09)	0.001
**Spontaneous vertex delivery (n=87)**	38 (43.68)	49 (56.32)	0.001
**Cesarean section (n=63)**	41 (65.08)	22 (34.92)	0.122
**Prolonged rupture of membranes (n=57)**	33 (57.89)	24 (42.11)	0.892
**Meconium staining (n=15)**	11 (73.33)	4 (26.67)	0.147
**Maternal Fever(n=43)**	19 (44.19)	24 (55.81)	0.052
**Anemia at delivery(n=113)**	45 (38.17)	68 (61.83)	0.00

Emergency CS has also been identified as a risk factor having significant influence on birth asphyxia. Studies done by Milsom^[^^15^^]^ and Seyal^[^^16^^]^ noted similar association, that deliveries by emergency cesarean section and use of instruments have direct association with birth asphyxia.

 Maternal age at delivery did not appear to be significant in our study as 54% of mothers were between 18 to 27 years of age and 46% were between 28 to 37 years of age. This was similar to the results reported from Sweden^15^. But maternal age more than 35 years is a risk factor associated with birth asphyxia in many studies. A possible explanation could be as vast majority of our patient group came from a low income, illiterate segment of population, many women were unaware of their exact age and the age stated was an approximation with a tendency towards younger age.

 In our study 112 (57%) cases of birth asphyxia occurred in para 1-3, whereas 84 (43%) in para more than 4, a study by Sayel^[^^16^^]^ described similar relation.

 In our study, PROM (>18 hours) was seen in 29% and pyrexia during delivery (>100 Fahrenheit) was seen in 22%. Both these factors are implicated with birth asphyxia as seen by Majeed^[^^5^^]^ et al in their study “risk factors of birth asphyxia” where they found relation of 24% and 20% respectively for prolonged rupture of membranes and maternal pyrexia.

 Meconium aspiration was seen in 7.6% cases and multiple births in 5.6% which is comparable to a local study^[^^5^^]^ where 9.6% and 4.8% of both these factors respectively are described.

 Gestational age of neonates did not appear to be significant risk factor in this study, as we excluded all preterm births less than 37 weeks and were only interested for the perinatal risk factors associated with birth asphyxia in term neonates. However, premature infants are reported in literature as being more prone to ischemic injuries of the white matter. These babies are more likely to have several other potentially fatal problems compared to term infants, since we only included term neonates, that could be explanation that no relation could be found.

 Maternal anemia at time of delivery was seen in 58% cases of birth asphyxia, maternal anemia is a significant risk factor for asphyxia, probably because of intrapartum hypoxia, and this observation is similar to Majeed et al^[^^5^^]^ where 60% of mothers were found anemic at time of delivery.

 Prolonged second stage of labor and birth asphyxia have strong correlation, reported in 72% of cases, improving awareness with easy access to a health services at delivery may play a role in reducing the incidence of birth asphyxia, as an average Pakistani woman is small sized therefore the chances of cephalo pelvic disproportion and prolonged labor leading to birth asphyxia is a possibility. Chishty^[^^12^^]^ et al noted similar relation.

 A study in India showed that traditional birth attendants were able to recognize signs of asphyxia, but were unable to deal with it^[^^11^^]^. Training program targeting at traditional birth attendants may be of benefit in decreasing birth asphyxia in our country where only 31% of deliveries are attended by skilled health personnel.

 There are certain limitations of this study as it is hospital based study on term neonates where majority of births were attended by qualified personnel, this doesn’t reflect exact epidemiology and associated risk factors prevalent in the community, where more than 70% of births are attended by traditional birth attendants. Strong point of the study is that it highlighted major pitfalls in our set up or any tertiary care hospital for instance and there is always room for improvement and emphasis must be on the importance of development of preventive strategies to reduce the burden of birth asphyxia. That will only be possible by imparting health education, better perinatal and obstetric care facilities at a tertiary care hospital. However, observations made in this study can help in planning larger population-based studies to confirm and target the risk factors of perinatal asphyxia.

## Conclusion

There is nothing more tragic for a normally developed fetus then to sustain cerebral anoxia during the last hours of perinatal life. This results in death in most cases and if escaped, have to live with a major handicap or lifelong disability. Since it is a preventable problem and long term neurological sequels are almost untreatable once asphyxia set in, there is no denying the fact that preventive strategies must be built to reduce the burden of birth asphyxia. Early identification of high-risk cases with improved antenatal and perinatal care can decrease such high mortality.

 We need to bring major reforms in our health delivery system. A properly trained person in neonatal resuscitation, preferably a pediatrician, should be available during high risk deliveries. Neonatal life support courses should be made mandatory for personnel involved in newborn deliveries and care. Early antenatal recognition and practice of referral to tertiary care hospital of high risk pregnancies will give better outcomes.
